# The potential of virtual healthcare technologies to reduce healthcare services’ carbon footprint

**DOI:** 10.3389/fpubh.2024.1394095

**Published:** 2024-05-16

**Authors:** Kim Usher, Jen Williams, Debra Jackson

**Affiliations:** ^1^Faculty of Medicine and Health, University of New England, Armidale, NSW, Australia; ^2^New England Virtual Health Network (NEViHN), Armidale, NSW, Australia; ^3^School of Nursing, Faculty of Medicine and Health, University of Sydney, Sydney, NSW, Australia

**Keywords:** telehealth, planetary health, virtual healthcare, carbon emissions, digital healthcare, virtual health education, climate change

## Abstract

The COVID-19 pandemic demonstrated the potential to reduce our carbon footprint especially by reducing travel. We aim to describe healthcare and health education services’ contribution to the global climate emergency and identify the need for increased use of virtual health service delivery and undergraduate/postgraduate education to help reduce the impact of health service and health education delivery on the environment. Health care services, as one of the largest contributors to carbon emissions, must take steps to rapidly reduce their carbon footprint. Health services have unfortunately paid little attention to this issue until recently. Virtual healthcare and education have a valuable role in transition to a net carbon-zero outcome. Given the increasing use of and satisfaction with virtual health services such as telehealth, and the increase in virtual education opportunities, it is important that a concerted effort is undertaken to increase their use across health services and education in the future.

## Introduction

### The potential of virtual healthcare technologies to reduce healthcare services’ carbon footprint

Healthcare services have become one of the largest contributors to greenhouse gas emissions (GHG) globally accounting for 4.4% of net GHG emissions (1, 2) as modern medical technology has become carbon emission intensive (3). Unfortunately, the healthcare sector continues to contribute significantly to GHGs (4). In fact, the healthcare sector has a significant and growing carbon footprint through energy consumption, transport, and product manufacture, use, and disposal (2). Unfortunately, healthcare services have only recently taken notice of this issue (2), and thus lag other sectors in taking action to reduce their climate impact (5). Tertiary education institutions, where most health professional education is delivered, are also high contributorus to GHGs mostly through travel, electricity and water consumption, and paper usage (6). Given the need for most health students to travel to attend mandatory clinical placements, which for some students amounts to large distances, is a growing concern.

Given the serious impacts of climate change on human and animal health and on the environment, it is timely to reflect on the carbon footprint of health and health education services and take action to reduce this footprint. It is paradoxical that the health sector, responsible for improving health outcomes, also directly contributes to poor health outcomes through excessive carbon emissions. The COVID-19 pandemic dramatically changed the way healthcare services and health education are delivered including an increased use of virtual technologies, especially telehealth, in place of face-to-face appointments and online teaching in place of face-to-face teaching. Telehealth services, which include telephone and video consultations as well as digital monitoring devices, also have benefits for the consumer. Travel costs and time away from home/work/family are reduced, access to services, especially specialist services for people living in rural and remote areas, is enhanced, chronic conditions are better managed as telehealth makes regular follow-up possible, wait times to see specialists are reduced, readmissions are decreased, and exposure to other patients and the general public in waiting rooms and during travel is reduced (7, 8). In addition, given that evidence suggests virtual student education has similar outcomes to real-life clinical placements (9), efforts need to be made to increase these opportunities for students.

## Virtual health as a strategy to reduce the health service carbon footprint

Among the contributors to health’s carbon footprint is the use of road and air travel for patients to attend face-to-face healthcare consultations, and travel by staff to consult with patients in rural and remote locations including their home, and to attend meetings (4). Experiences arising from the COVID-19 pandemic provide important data that demonstrates how health services’ contribution to GHGs can be reduced. During the pandemic, the use of virtual health technologies such as telehealth (both video and telephone consultations) increased dramatically (10) and subsequent research has shown that virtual healthcare not only offers a satisfactory alternative to face-to-face consultations but also reduces the need for patients (and providers) to travel (11). Tsagkaris et al. (3) claim important learnings from the COVID-19 pandemic include that virtual healthcare technologies such as telehealth offer effective alternatives to face-to-face healthcare visits, reducing the need for patients to travel to attend health consultations and reducing environmental costs (4). Reducing unnecessary patient travel has been identified as an effective way of reducing the carbon footprint of health services (12). Telehealth services such as video and telephone consultaions and in-home digital monitoring devices offer a means of achieving this outcome.

Telehealth (also called telemedicine) is a recent development. It describes various forms of remote consultation that can encompass a range of digital tools including both video and telephone consultations and digital monitroring devices (13, 14). A recent review highlighted issues around accessibility and establishing a therapeutic relationship as among the major concerns in the use of video abd telephone telehealth platforms (14). There is evidence to suggest that video supported telehealth is preferred over telephone consultations and that telehealth is rated as more effective than telephone consultations by patients (15). Telephone consultations however generally require a lower level of digital literacy and may represent enhanced accessibility over other methods for remote consultations (14). Overall, telehealth has been viewed satisfactorily by patients (16, 17) with benefits including less travel, less time away from home and work, and lower costs (18).

Other virtual healthcare innovations such as digital devices (including wearable and implantable devices, remote imaging, and mobile apps), allow patients to be assessed and monitored effectively at home (1, 4, 19, 20). These alternative means of health assessment and monitoring, and healthcare delivery offer potential to reduce the carbon footprint of healthcare services, by reducing the need for patients and staff to burn fossil fuels while traveling to attend healthcare consultations and home or clinic visits (18). There is also a likely reduction in the scope of emissions, associated with disposables and consumables (12), with remote consultations. The environmental benefits of the reduction of travel were demonstrated during the COVID-19 pandemic when an 8.8% reduction in global emissions reported in the first half of 2020 compared to the same period of 2019 (21). While there have been some concerns raised about the use of virtual health technologies acceptance, use of virtual health is increasing (18, 22).

## Potential for virtual health undergraduate and postgraduate student clinical placements to help reduce the carbon footprint

There is an urgent need to develop more sustainable health education practices to help reduce climate change. Unfortunately, it appears that while health educators have the knowledge to do this, they lack the pedagogical expertise to teach this information (10). To address this issue, a recent Australian study identified the need to embed sustainable health education, policy and practice using “…an evidence based, interdisciplinary whole health and tertiary education approach” (p. 325) (23). Most health professional training programs require students to undergo workplace training under the supervision of an experienced educator/clinician. Nursing regulatory bodies in the United Kingdom and United States have already supported the replacement of some face-to-face clinical placements with virtual simulation (24). Virtual simulation activities have the potential to reduce the need for travel to attend clinical placements hence reducing GHG emissions and reducing student costs.

Evaluations of virtual simulation placements in nursing are positive with a recent study finding that the virtual clinical replacement experience was statistically significant reporting greater confidence in areas such as patient safety, communication, and leadership, as well as greater perceived support in the workplace (9). The use of virtual simulation with medical students has also been positive with students reporting being better prepared for the clinical environment (25). Virtual technologies offer an adjunct or even an alternative to clinical placements that can help reduce the health services’ carbon footprint.

## Evidence that reduced travel leads to reduced GHG emissions

A Spanish study by Morcillo Serra et al. (26) found that 640,000 digital consultations and 3,060,000 medical reports downloaded remotely by patients during the COVID-19 pandemic in 2020 avoided an estimated 6,700 net tons of CO^2^ emissions. That study demonstrates the potential reduction in GHG that can be achieved through the increased use of virtual health. Numerous studies have been recently conducted that demonstrate similar reductions if virtual health was used as an alternative to traditional consultations (4, 27). While there is the potential for increased GHG emissions associated with use of electricity used to power increased digitalisation of healthcare services, often overlooked in many studies conducted to date, these increases were found to be far less than those associated with patient and staff travel (4, 27). In one study that did include assessment of energy used to run equipment for telehealth, it was estimated that telerehabilitation services resulted in carbon cost savings when the patient travels over 7.2 kms to attend the appointment (28). It is important to remember that the different virtual technologies use differing amounts of GHGs with video enhanced telehealth more GHG emission intensive than telephone calls (4). The review by Purohit et al. (4) reveals the importance of considering the medical specialty, geography, and time. It seems that the higher the level of specialization corresponds with a greater reduction in travel, since specialized centres serviced a wider geographic region. For example, studies of telephone consultations in place of face-to-face visits have been evaluated. A study of post-renal transplant services telephone follow-up appointments for 30 patients resulted in a saving of 39.3 km travel equating to a saving of 8.00 kg CO_2_ per consultation (29), and a study of pre-surgical telephone consultations in Texas, where large distances were traveled to the one specialist service, resulted in carbon footprint reductions of 271 kg CO_2_ per consultation (30). Similarly, evaluations of video conference CO_2_ savings have also been positive. For example, the use of videoconferencing for telerehabilitation in Sweden which included the energy consumption of the equipment as well as travel savings demonstrated that 238 apointments resulted in a saving of 82,310 km giving a range of 87.5–175 kg CO_2_ per consultation (28).

## Need for urgent action

A recent editorial (31), identified the need for urgent action and proposed we are facing a global health emergency. Health services must not only deliver healthcare to those made ill from the climate crisis, but also radically reduce their own emissions (2). Health professionals and educators must take an urgent role in developing and utilising strategies that help reduce the health services’ and health education carbon footprint. In Australia, where health travel is extensive due to the size of the country, it is even more urgent to tackle this wicked problem. Outside of emergency responses, telehealth has shown to have similar outcomes to standard consultations for many health conditions including diabetes and cardiac conditions (27). Greater implementation of virtual healthcare and education technologies offers an opportunity to reduce the need for travel and in turn, reduce the healthcare carbon footprint. Efforts are needed to ensure research approaches, education and policy are developed to facilitate greater use and evaluation of virtual healthcare services. Similarly, educators need to look for opportunities to reduce the need for travel, especially travel over large distances, for students to attend clinical placements or fieldwork requirements. The potential for carbon emission reductions in this area is huge.

## Conclusion

Health services, as every sector of society, have a responsibility to take action to reduce the impacts of humans on the environment. Virtual healthcare and education services have a valuable role in transition to net carbon-zero healthcare/education services for the future. As the current evidence suggest a strong relationship between carbon footprint reductions and average distance travelled, countries with larger distances to travel for face-to-face consukltations may benefit more by enhancing their use of telehealth services for patient consuktations where possible. Given the increasing use of and satisfaction with virtual health services such as telehealth, it is important that there is a concerted effort to increase their use across health services. It is also imperative that health education adopts ways to improve student/educator awareness of the need to reduce the health carbon footprint and adopts virtual health education practices that have the potential to further reduce the current health education carbon footprint. Given the need to reduce the GHGs emitted by health services and education services, health professionals and educators have a pivotal role in building healthier, more equitable and sustainable health services and education by adopting practices that have a lesser impact on the environment. Greater efforts are needed to ensure research approaches, education, and policy are developed to support the increased use of virtual healthcare services and undergraduate and postgraduate student clinical placements, and to ensure ongoing evaluation of these services as they are integrated into mainstream healthcare and tertiary education.

## Data availability statement

The original contributions presented in the study are included in the article/supplementary material, further inquiries can be directed to the corresponding author/s.

## Author contributions

KU: Conceptualization, Investigation, Writing – original draft, Writing – review & editing. JW: Conceptualization, Investigation, Writing – original draft, Writing – review & editing. DJ: Writing – original draft, Writing – review & editing.
